# Natural and artificial selection and suffering and well-being

**DOI:** 10.3389/fgene.2014.00393

**Published:** 2014-11-12

**Authors:** Jeroen van Rooijen

**Affiliations:** Retired: Former Poultry Behavior Researcher Center for Applied Poultry ResearchHet Spelderholt, Beekbergen, Netherlands

**Keywords:** suffering, well-being, welfare, natural selection, artificial selection, domestication

## Introduction

In this essay I assume the existence of subjective states of suffering and well-being (welfare is often used as a synonym for well-being) in animals (Van Rooijen, 1981). For my opinion about the relation between suffering and well-being and the study of behavior, see Van Rooijen (1997). This article will compare suffering and well-being due to natural selection and suffering and well-being due to artificial selection. As far as I know this comparison is hardly made in literature.

Lorz (1973, cited in Van Putten, 1981) has defined well-being of an animal as “Living in harmony with the environment and itself, both physically and psychologically.” In a healthy animal all bodily processes are tuned in with each other. We may say that the animal is in harmony with itself (its physiology is in harmony). However, healthy animals may suffer too. For instance, because their husbandry conditions are (or have been) too remote from the natural environment to which their wild counterpart has been adapted, as may be indicated by stereotypic behavior (Van Rooijen, 1984).

During its phylogeny a species is adapted to its own natural environment. For instance, a polar bear is adapted to a polar environment. When the situation is not too extreme we may take the well-being of a polar bear in its natural situation for granted. However, a tropical bird will suffer under polar conditions. We may say that a polar bear is in harmony with a polar environment and a tropical bird with a tropical environment. In a similar way a camel is adapted to (in harmony with) an environment with little water while a whale is adapted to (in harmony with) an environment with plenty of water. When an animal is not in harmony with itself and/or with its environment we may assume that it suffers. When an animal is in harmony within itself and with its environment we may assume that it experiences well-being.

We may distinguish predators and prey animals. Companion animals (cats, dogs, guinea pigs, rabbits, birds, fish, reptiles, etc.) are sometimes predators and sometimes prey animals. Farm animals are almost exclusively prey animals (horses, cows, goat, sheep, swine, hens, turkeys, rabbits, an exception are fur animals as minks and foxes). Laboratory animals may also be one of both types: dogs and cats are predators, rabbits, mice and rats are prey animals.

## Suffering and natural selection

Wild animals may have super normal preferences, for instance, for a larger than normal egg size (Tinbergen, 1948, cited Hinde, 1966) or particular characteristics in their sexual partner. Such preferences are not fulfilled in nature because there are also other selection pressures. For instance, it would not be possible to breed too large eggs properly. Perhaps peacock females prefer males with even longer tails than in nature but such males become too easily predated. Perhaps super normal preferences may decrease the well-being, which shows that the harmony in nature is not always perfect.

Wild animals in nature may suffer from a lack of resources as territories or nesting sites. However, such a lack of resources does not result in stereotypic behavior. Perhaps the animals are in some degree adapted to such situations. In wild animals in nature variation will emerge. The genetic basis of this variation is the point of application for natural selection. Less adapted variants may suffer, but even individuals with a higher fitness may have a decreased well-being (for instance, because they have to work harder to provide food for their young than individuals with a lower fitness).

In equilibrium situations parents are on average only replaced in the next generation. Most offspring die before they reproduce. Natural selection may work by accidents and/or starvation and/or disease. This may cause suffering before the animal dies. In equilibrium situations a continuous pressure by predators on prey animals is present. As a result, in nature, prey animals may suffer more from acute stress during predation than from chronic stress. I do not know whether predators are often killed by other predators. If not this may result in chronic suffering due to hunger and disease. However, they do not seem to perform stereotypic behavior. Further, wild animals have to cope with disturbances in nature caused by man. This may cause suffering too.

## Well-being and natural selection

Dawkins (1976) mentions Young (1975), who has pointed out that genes have to perform a task analogous to prediction. Dawkins writes that polar bear genes predict that the future of the unborn polar bear is going to be a cold one: “They do not think of it as a prophecy, they do not think at all: they just build a thick coat of hair, because that is what they have always done before in previous bodies, and that is why they still exist in the gene pool. They also predict that the ground is going to be snowy, and their prediction takes the form of making the coat of hair white and therefore camouflaged.” (White hairs are hollow and, therefore, also a good isolation.) Animals also have a prediction about useful behavior patterns. For instance, a dog often circles around before it lies down. The function of this behavior is to create a lying place. The dog still “predicts” that it will live in an environment with vegetation. In nature animals are, thus, in some degree in harmony with themselves and their environment (as indicated by the absence of stereotypic behavior). Most of the time we may therefore take their well-being for granted. Natural selection leads to a further fine tuning of the traits of a wild species to its natural environment. That means that the wild animals become more adapted to their natural environment and this increases their well-being.

## Suffering and artificial selection

To understand the situation of domestic animals under artificial conditions it is helpful to realize that these animals still predict that they will live in their natural environment. [This explains why many domestic species (horses, swine, dogs, cats, hens, etc.) easily become feral.] Not only during the ontogeny but also during adult life the environment is different from the one the animals predict; both may result in chronic stress. The ontogeny may also not be in harmony with the adult environment. This brings the animal even more in disharmony (Van Rooijen, 1982). Stereotypic behavior may occur among cats and dogs under artificial conditions (e.g., animals in shelters, animals left alone at home, animals under laboratory conditions) and among minks and foxes under intensive conditions. In farm animals under intensive conditions chronic stress is common. Perhaps, chronic stress is similar to the chronic stress experienced by psychiatric patients (Van Rooijen, 1983). Acute stress may occur in cats and dogs (e.g., visits to the veterinarian). Acute stress may also occur in farm animals. Especially during the catching of animals, but also during weaning, regrouping, castration, injections, sexing, wing clipping, beak trimming, teeth clipping, nail clipping, tail clipping, comb clipping, transportation, slaughtering, etc. Domestic animals may also be in disharmony because of their genotype:

*Hybrids*. Domestic species are sometimes the result of hybridization between different species or subspecies adapted to different niches. Such hybrids are sometimes not in harmony within their own physiology (for instance, neurology). Well-known examples are the hybrids between love bird species made by Dilger (1962). The parental species have different methods to transport nesting material to the nest. The hybrids are frustrated because they are hardly able to combine these methods successfully. This may decrease their well-being. More fundamental seems the frustration due to contradictory tendencies in hybrids between solitary and social species (e.g., hybrids of tigers and lions).
*Inbreeding*. When an inbreeding population is founded by a few individuals the number of genes present in such a population is only limited (the founder effect). This is exaggerated by genetic drift, especially when the population goes through bottlenecks. This results in a greater risk that individuals are homozygote for recessive genes that cause genetic diseases. This may decrease their well-being.
*Selection for deviant traits*. Many breeds are based on deviant individuals (sometimes animals with a mutation). This deviant trait is often exaggerated by selection. A deviant trait may hamper the normal functioning of an individual. The physiology of such individuals may less be in harmony. In such breeds selection toward the wild genotype may improve well-being. Hybridization of lines with the wild genotype may, therefore, often help to increase the well-being of the offspring. However, such hybridization does not help in all cases. For instance, severe feather pecking is an abnormal behavior that causes much suffering among flock mates. Indeed, wild bankiva fowl do not perform this behavior in nature but they do under suboptimal artificial conditions. In such cases hybridization with the wild genotype is not helpful to decrease suffering under suboptimal artificial conditions.
*Selection for a few traits*. Even if a trait is not that deviant that it hampers well-being, selection on a few traits may have the result that the physiology is no longer in harmony. This happens for instance, when hens are selected for larger eggs but are not selected for a larger cloaca width (Van Rooijen, 1983). Also animals selected for a higher weight are not in harmony when they are not also selected for stronger legs.
*Selection for another generation*. Broilers are selected for a large appetite. However, broiler breeders are restrictedly fed. This may imply that these animals suffer from chronic hunger.
*Absence of selection pressures*. When the maintenance of traits costs energy it is likely that such traits will become rudimentary when selection pressures on the trait are no longer present. This explains why animals on islands without predators may become tame. Birds and insects on such islands may, for this reason, lose their ability for flying. It also explains why some fish species living in caves where no daylight ever enters have become blind. Internal parasites like tape worms may in many respects rely on the constant conditions provided by their host. Therefore, many of the capacities which were present in the ancestors of tape worms became rudimentary during the phylogeny. Man provides farm animals also with water, food, a constant temperature, etc. This may make farm animals also lose capacities. Therefore, we may assume that animals completely adapted to the conditions of intensive husbandry become similar to internal parasites like tape worms (Van Rooijen, 1983).

## Well-being and artificial selection

Under domestic conditions, compared with natural conditions, well-being may be improved because of hygiene, veterinary care and protection against predators. Well-being may also be improved by unconscious and conscious selection. Animals which are more tolerant toward artificial conditions are less stressed under such conditions and may, therefore, have access to food and a higher fitness. This may explain why some wild species suddenly successfully invade cities. Such species become more or less domesticated. Also animals that are more tolerant toward sexual partners may have a higher fitness. This explains why in zoos reproduction of particular species (e.g., tigers) suddenly becomes successful. Such species also become more or less domesticated. This increases their well-being.

## Concluding remarks

1. For predators and prey animals we may conclude that the difference in acute stress in nature and under domestic conditions is not obvious.
2. Stereotypic behavior may be an indication of chronic stress. Such behavior is more common under intensive than under traditional conditions. In nature stereotypic behavior indicating chronic stress seems absent.
3. Animals under intensive and under traditional conditions may suffer because their physiology is not in harmony.
4. Natural selection leads to a further fine tuning of the traits of a wild species to its natural environment, therefore, natural selection will increase well-being in the long run.
5. Artificial selection may increase suffering but may also increase well-being. Artificial selection seems to be an important tool to decrease suffering and increase well-being in domestic animals under artificial conditions. I am of opinion that we need selection that results in animals that are more in harmony with themselves and with their environment.

### Conflict of interest statement

The author declares that the research was conducted in the absence of any commercial or financial relationships that could be construed as a potential conflict of interest.
